# Scaling the Equipment and Play Area in Children’s Sport to improve Motor Skill Acquisition: A Systematic Review

**DOI:** 10.1007/s40279-015-0452-2

**Published:** 2016-01-16

**Authors:** Tim Buszard, Machar Reid, Rich Masters, Damian Farrow

**Affiliations:** Institute of Sport, Exercise and Active Living/College of Sport and Exercise Science, Victoria University, PO Box 14428, Melbourne, VIC 8001 Australia; Tennis Australia, Private Bag 6060, Richmond, VIC 3121 Australia; Australian Institute of Sport, PO Box 176, Belconnen, ACT 2617 Australia; Te Oranga School of Human Development and Movement Studies, Faculty of Education, University of Waikato, Hamilton, New Zealand

## Abstract

**Background:**

This review investigated the influence of scaling sports equipment and play area (e.g., field size) on children’s motor skill acquisition.

**Methods:**

Peer-reviewed studies published prior to February 2015 were searched using SPORTDiscus and MEDLINE. Studies were included if the research (a) was empirical, (b) involved participants younger than 18 years, (c) assessed the efficacy of scaling in relation to one or more factors affecting skill learning (psychological factors, skill performance and skill acquisition factors, biomechanical factors, cognitive processing factors), and (d) had a sport or movement skills context. Risk of bias was assessed in relation to selection bias, detection bias, attrition bias, reporting bias and other bias.

**Results:**

Twenty-five studies involving 989 children were reviewed. Studies revealed that children preferred using scaled equipment over adult equipment (*n* = 3), were more engaged in the task (*n* = 1) and had greater self-efficacy to execute skills (*n* = 2). Eighteen studies demonstrated that children performed skills better when the equipment and play area were scaled. Children also acquired skills faster in such conditions (*n* = 2); albeit the practice interventions were relatively short. Five studies showed that scaling led to children adopting more desirable movement patterns, and one study associated scaling with implicit motor learning.

**Conclusion:**

Most of the studies reviewed provide evidence in support of equipment and play area scaling. However, the conclusions are limited by the small number of studies that examined learning (*n* = 5), poor ecological validity and skills tests of few trials.

## Key Points

**Table:** 

Scaling constraints in the environment (equipment and play area) allows children to play sport in a manner that more closely represents the adult game.
Evidence suggests that scaling is an effective strategy to enhance skill performance and this seems to aid learning.
Sports authorities should aim to create environments in junior sport that simplify skill performance whilst maintaining perception–action couplings akin to the adult game.

## Introduction

**Fig. 1:**
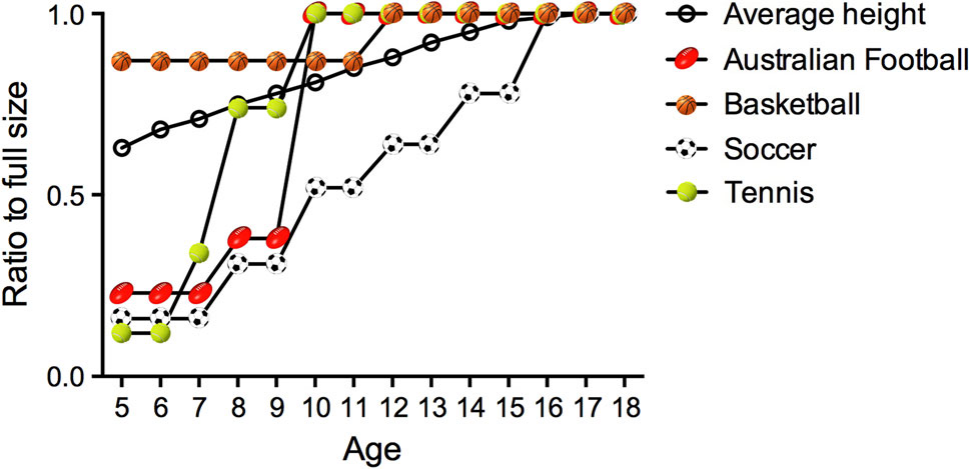
Recommended play area (*court*, *pitch* or *oval size*) for different age groups across four popular sports. These guidelines were based on recommendations by major sports organizations across the world: International Basketball Federation (*basketball*), International Tennis Federation (*tennis*), The Football Association (*soccer*) and the Australian Football League (*Australian Football*). Play area has been standardized to a ratio out of 1, with 1 representing a full-size (adult) play area. The play area ratios are mapped against the average height of children (boys and girls combined) from 5 to 18 years. Soccer appears to be the only sport in which the recommended play area dimensions increase at a similar rate to children’s height. The other sports recommend that children play on adult-sized dimensions from approximately age 10 years onwards

The value of scaling sport for children is patently clear. Consider a 7-year-old playing basketball with a full size ball and a basket at the same height as used for an adult, or a 6-year-old playing tennis on a full size court with a ball that bounces above the head. In both circumstances, children are likely to experience difficulty in completing the task successfully. Despite the logical benefits of modifying the constraints imposed on children during sport, our knowledge of how these modifications may influence the acquisition of their skills is limited. Moreover, in using stature as a proxy for scaling, it seems likely that the guidelines prepared by most sports authorities are comparatively more challenging for young children than adults (see Fig. 1). Our aim was to systematically review the scientific literature that informs how scaling key constraints in children’s sport—equipment and play area—influences subsequent acquisition of motor skills by children.

According to the constraints-led approach, skill acquisition is a process of self-organization that is dependent upon constraints imposed on the system [1–3]. The constraints can be internal or external features that define the boundaries within which the human neuro-musculoskeletal system(s) must operate. Specifically, Newell [2] defined three categories of constraints: organismic (the individual’s physical and psychological characteristics), environmental (the external forces surrounding the performer) and task-related (the rules and goals of the task and the equipment used). As such, optimal movement patterns are considered to emerge from the convergence of organismic, task and environmental constraints. For example, the scaling of equipment (task constraint) may provide young children, who often lack the strength required to use adult equipment proficiently (organismic constraint), the opportunity to perform the necessary skills and therefore find the optimal movement solution when playing in a match, particularly when external conditions, such as weather, are less favorable (environmental constraint). In doing so, this may facilitate the coupling of perception–action processes, which is considered essential for coordinated movement patterns [1].

This review will focus on four inter-related themes that influence skill. First, we review literature that examines the relationship between modified sport for children and psychological factors such as self-efficacy and engagement with the task. Second, we discuss empirical evidence that links scaling in children’s sport with enhanced skill performance and skill acquisition. In particular, we will focus on the benefits of scaled equipment for teaching skills to children. Additionally, we will discuss the influence that modified equipment and reduced play area have on practice and match conditions compared with full-size equipment and adult-sized play areas. Third, we will look at scaling from a biomechanical perspective. We specifically explore whether scaling the equipment and play area for children leads to the development of more biomechanically efficient movements and, logically, a reduced risk of injury. Fourth, we investigate a recent body of literature that examines the interaction between equipment modification and cognitive processes. This is achieved by critiquing whether scaling equipment to simplify a task encourages implicit motor learning and/or whether the use of equipment that increases task difficulty promotes explicit motor learning.

## Methods

### Data Sources

Keyword searches identified articles from two electronic databases: SPORTDiscus and MEDLINE (11 February 2015). The following terms were used: (*equipment* OR *ball* OR *racquet OR racket* OR *bat* OR *golf club* OR *goals* OR *play area* OR *court* OR *field*) AND (*child* OR *children* OR *youth* OR *junior*) AND (*sport* OR *tennis* OR *golf* OR *volleyball* OR *basketball* OR *softball* OR *baseball* OR *netball* OR *football* OR *soccer* OR *gymnastics* OR *cricket* OR *rugby* OR *athletics* OR *hockey* OR *swimming* OR *water polo*) AND (*modified* OR *scaling* OR *scaled* OR *mini*). Only academic journal articles were included in the search.

### Inclusion/Exclusion Criteria

**Fig. 2:**
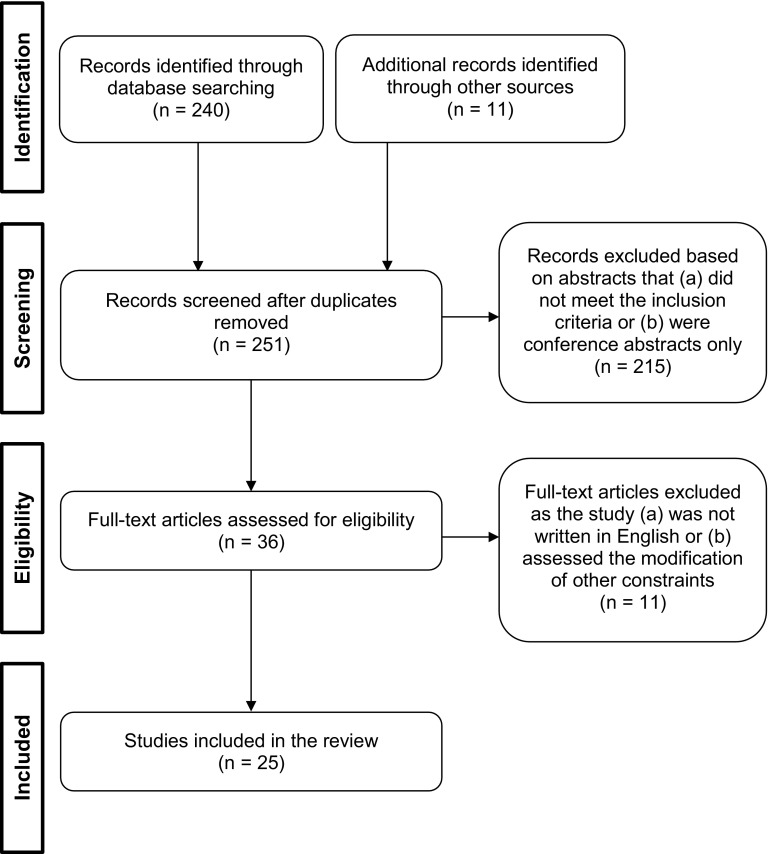
PRISMA flowchart representing each stage of the review process (adapted from Moher et al. [4])

A study was included in this review if it met the following criteria: (a) the research was empirical (qualitative or quantitative evidence); (b) the focus was on children and/or youth aged younger than 18 years; (c) the study variables included measures that assessed the efficacy of scaling in relation to motor skill learning (psychological factors, skill performance and acquisition factors, biomechanical factors, cognitive processing factors); and (d) the context was sport or movement skills. The initial search yielded 240 potential studies (see Fig. 2). A total of 226 studies were excluded for the following reasons: six were duplicates, 206 did not meet the inclusion criteria, five were not written in English, three were conference abstracts only, and six examined the influence of modifying other constraints (e.g., the rules) as opposed to modifying equipment or area of play. In addition to the database search, a further 11 articles meeting the inclusion criteria were found by searching reference lists. Overall, 25 studies were examined in this review.

### Risk of Bias Assessment

Many systematic reviews adopt a protocol for assessing the quality of studies using standardized assessments. However, such assessment tools are scarce for skill acquisition research. Consequently, we opted to follow the guidelines for healthcare research, in which the *Cochrane Handbook for Systematic Reviews* discourages the assessment of study quality in favor of assessing the risk of bias within each study [5]. The Cochrane Collaboration tool for assessing risk of bias addresses six types of bias that can occur in research, with five of these being relevant to skill acquisition studies:^1^ (1) selection bias, (2) detection bias, (3) attrition bias, (4) reporting bias, and (5) other bias. *Selection bias* refers to the inadequate generation of randomized groups or randomized order of conditions; *detection bias* occurs when the outcome assessors (e.g., subjective assessment of movement technique or match play performance) have knowledge of the allocated intervention; *attrition bias* refers to the amount of missing data and how it is treated; *reporting bias* is due to the selective reporting of outcome data; and *other bias* occurs for issues not elsewhere covered.

There were two parts to the assessment. First, information related to each category of bias was gathered and entered into a table for each study reviewed. This information was typically in the form of verbatim quotes from the article. Knowledge of study protocols (e.g., via correspondence with lead authors of relevant studies) was also used as evidence. This was completed by the lead author (TB). The second part required judgments to be made regarding the risk of bias based on the information provided. The risk of bias was categorized as either low, high or unclear (i.e., insufficient information to conclude whether the risk of bias was low or high), with judgments based on the guidelines provided by the Cochrane Collaboration tool (see also Higgins et al. [6]). Two reviewers (TB and DF) made independent judgments about each study considered for review. The third and fourth reviewers (MR and RM) were consulted for any discrepancies that arose.

## Results and Discussion

### Risk of Bias Assessment

Of the 25 studies reviewed, 21 were deemed to have low risk of bias in all five categories (see Table 1). Two studies were considered to have high selection bias, as the participants were not randomly allocated to practice groups [7] or the conditions of testing favored scaled equipment [8]. In the latter case, testing involved five trials with a women’s basketball followed by five trials with a junior basketball for every participant, thereby creating a potential learning effect that favored the junior ball. For two studies, it was unclear whether the risk of detection bias was high or low, because technique was subjectively assessed by “observers,” but it was unclear whether the observers were independent from the research team and/or blinded to treatment allocation [7, 9]. However, it must be noted that Hammond and Smith [7] did not explicitly state whether it was hitting technique or hitting accuracy that was assessed in their skills tests. Intra- or inter-reliability was also not obtained in these two studies. None of the studies reported missing data and only one was considered to be at high risk for selective reporting [10]. Specifically, Pellett et al. [10] discussed the skill learning advantages when practicing with the modified volleyball, despite no supporting evidence from the skills testing results. The Pellett et al. [10] study was also deemed to have another bias in its design, as the skills tests were only performed with the regulation volleyball; thus, children who practiced with the lighter volleyball during the study were likely to be disadvantaged in the skills test. Indeed, this may explain the lack of differences observed in this study. The remainder of this article discusses the findings of the reviewed studies in the context of these limitations.

**Table 1:** Risk of bias assessment

Sport	References	Random sequence generator (selection bias)	Blinding of outcome assessment (detection bias)	Incomplete outcome data (attrition bias)	Selective reporting (reporting bias)	Other bias
Basketball	Szyman et al. [8]	High	Low	Low	Low	Low
	Arias [11]	Low	Low	Low	Low	Low
	Arias [12]	Low	Low	Low	Low	Low
	Arias et al. [13]	Low	Low	Low	Low	Low
	Arias et al. [14]	Low	Low	Low	Low	Low
	Arias et al. [15]	Low	Low	Low	Low	Low
	Arias et al. [16]	Low	Low	Low	Low	Low
	Regimball et al. [9]	Low	?	Low	Low	Low
	Chase et al. [17]	Low	Low	Low	Low	Low
	Satern et al. [18]	Low	?	Low	Low	Low
Cricket	Elliott et al. [19]	Low	?	Low	Low	Low
Fundamental skills						
Throwing	Burton et al. [20]	Low	Low	Low	Low	Low
Catching	Isaacs [21]	Low	Low	Low	Low	Low
Multiple sports: bowling, basketball, throwing and baseball	Wright [22]	Low	Low	Low	Low	Low
Tennis	Timmerman et al. [23]	Low	Low	Low	Low	Low
	Buszard et al. [24]	Low	Low	Low	Low	Low
	Buszard et al. [25]	Low	Low	Low	Low	Low
	Kachel et al. [26]	Low	Low	Low	Low	Low
	Lee et al. [27]	Low	Low	Low	Low	Low
	Larson and Guggenheimer [28]	Low	Low	Low	Low	Low
	Farrow and Reid [29]	Low	Low	Low	Low	Low
	Hammond and Smith [7]	High	?	Low	Low	Low
	Gagen et al. [30]	Low	?	Low	Low	Low
	Elliott [31]	Low	Low	Low	Low	Low
Volleyball	Pellett et al. [10]	Low	Low	Low	High	High

*High* high risk of bias, *low* low risk of bias, *?* unclear whether the risk of bias was low or high on the basis of the information provided in the article

### Overview of Findings

The reviewed studies examined a total of 989 children, with most studies focusing on basketball (*n* = 343 children) and tennis (*n* = 313 children). As such, our discussion may appear to focus largely on these sports (see Table 2); however, we suspect that the findings can be generalized to a wide range of skills across multiple sports. We discuss the findings of the review in four sections: psychological factors, skill performance (and acquisition) factors, biomechanical factors, and cognitive processing factors. We acknowledge that several of the reviewed studies provide evidence related to multiple sections (e.g., both psychological factors and skill performance factors) and, therefore, some of our discussion crosses sections.

**Table 2:** Studies examining the influence of equipment scaling on children’s sport performance

Sport	References	Modification	Population studied	Primary outcome: positive (Y/N)	Main finding
Basketball	Szyman et al. [8]	Ball mass and diameter	11 years, disabled/wheelchair	Y	Children displayed more accurate shooting when using smaller and lighter basketballs from the 2 distances examined: 13 ft and 10 ft from the ring
	Arias [11]	Ball mass	9–11 years, intermediate	Y	The number of attempted lay-ups increased when children played with the lighter ball (440 g) compared with a regulation ball (485 g) and a heavier ball (540 g) during matches
	Arias [12]	Ball mass	9–11 years, intermediate	Y	Shot accuracy and shot efficacy was greater when playing with a lighter ball (440 g) compared with a regulation ball (485 g) and a heavier ball (540 g) during matches
	Arias et al. [13]	Ball mass	9–11 years, intermediate	N	No significant differences were found between 3 ball types (440, 485 and 540 g) for the number of attempted shots and number of successful shots from any distance during matches
	Arias et al. [14]	Ball mass	9–11 years, intermediate	Y	Children passed the ball more, displayed more pass receptions and dribbled more often when using a lighter ball (440 g) compared with a regulation ball (485 g) and a heavier ball (540 g) during matches
	Arias et al. [15]	Ball mass	9–11 years, intermediate	Y	Frequency of shot attempts and the number of successful shots were greater with the lighter ball (440 g) compared with a regulation ball (485 g) and a heavier ball (540 g) during matches
	Arias et al. [16]	Ball mass	9–11 years, intermediate	Y	Children experienced more one-on-one situations when playing with the lighter ball (440 g) compared with a regulation ball (485 g) and a heavier ball (540 g) during matches
	Regimball et al. [9]	Ball dimensions	10 years, beginners	N	No difference in performance (free-throw shooting) between ball types; however, performance was better for the particular ball that children preferred. 62 % of children preferred using the smallest ball and 45 % preferred using a ball that is smaller than the one they usually use
	Chase et al. [17]	Basket height and ball dimensions	6–7 years^a^	Y	Children were more successful when shooting to the lower basket (2.44 m) compared with the higher basket (10 ft). Self-efficacy was also higher when shooting to the lower basket (3.05 m). Ball size had no influence on shooting performance, but shot efficacy was greater with the smaller ball than the larger ball
	Satern et al. [18]	Ball mass and diameter, and basket height	12 years^a^	Y	Lowering the basket from 3 to 2.4 m resulted in a change in shooting trajectory for free-throw shots. However, there was no assessment of how this influences shooting accuracy. Ball size had no effect on movement kinematics
Cricket	Elliott et al. [19]	Pitch length	10, 12 and 14 years^a^	Y	Children in each age group bowled more accurately at a shorter pitch length. Under-11 and under-13 bowlers displayed techniques that were seemingly more prone to injuries when bowling on a full-length pitch as opposed to the shorter pitch. Under-15 bowlers displayed a similar technique on the full-length pitch as the shorter pitch
Fundamental skills					
Throwing	Burton et al. [20]	Ball diameter	5–1 years, beginners, and adults	Y	Children and adults displayed a regression in throwing patterns when the ball size increased to a diameter that was greater than the performer’s hand width
Catching	Isaacs [21]	Ball diameter	7–8 years^a^	Y	Children caught the smaller ball (6-in. diameter) with a more mature style than the larger balls (10-in. diameter)
Multiple sports: bowling, basketball, throwing and baseball	Wright [22]	Ball mass for all sports and baseball bat weight	7–8 years^a^	N	7-year-old girls were reported to perform better with lightweight equipment than heavyweight equipment during an assessment of skill 2 days following 1 practice session. Conversely, no differences were reported between equipment types for the 8-year-old girls. For boys, both 7- and 8-year-olds tended to perform better with heavyweight equipment^b^
Tennis	Timmerman et al. [23]	Court size and net height	9–10 years, skilled	Y	Lowering the net by 22 cm resulted in more winners, volleys and shots played at a comfortable height, and fewer shots played behind the baseline, which represents more aggressive play
	Buszard et al. [24]	Racquet length and ball compression	6–9 years, beginners	Y	Forehand performance (accuracy and technique) was best when using the lowest compression ball (25 % of standard ball, “red”) combined with a scaled racquet (19-in.). The ball had a greater influence on performance than the racquet
	Buszard et al. [25]	Racquet length and ball compression	9–11 years, beginners	Y	Forehand performance (accuracy and technique) was better when using a low compression ball (75 % of standard ball, “green”) combined with a scaled racquet (23-in.) compared with a standard ball and a full-size racquet
	Kachel et al. [26]	Ball compression	9–10 years, skilled	Y	When using the low compression ball (75 % of standard ball, “green”), as opposed to the standard ball, children played more balls at a comfortable height, approached the net on more occasions and had faster rallies
	Lee et al. [27]	Net height, target area, court size	9–10 years, beginners	Y	Constantly modifying the net height, target areas and court size to create a variable practice environment led to children displaying a greater number of movement clusters^c^ following 4 weeks of practice (600 forehands) compared with children who practiced repetitive drills with the same net height, target areas and court size
	Larson and Guggenheimer[28]	Ball compression and court size	7–9 years, intermediate	Y	Skills test performance was better when using a low compression ball (75 % of standard ball)^d^ on a scaled court compared with when using a standard ball on a full-size court
	Farrow and Reid [29]	Ball compression and court size	8 years, beginners	Y	Practicing on a full-size court with a standard ball resulted in negative learning relative to practice on a scaled court and/or with a low compression ball (<50 % of standard ball, “red”)^e^ after 5 × 30-min practice sessions. The court had a greater influence on learning than the ball
	Hammond and Smith [7]	Ball compression	5–11 years, beginners	N	No differences in tennis skills tests were present between a group practicing with a low compression ball (25 % of standard ball, “red”)^f^ and group practicing with a standard ball following 8 × 60-min practice sessions
	Gagen et al. [30]	Racquet length	4–10 years, beginners	N	Although every child had one racquet that they swung better than others, the characteristics of this racquet were not related to the child’s size or strength
	Elliott [31]	Racquet length	7–10 years, beginners	Y	The groups that practiced with the smallest racquets displayed superior performance on measures of tennis skill compared with the group that practiced with the larger racquet following 16 × 50-min practice sessions
Volleyball	Pellett et al. [10]	Ball mass	7th grade^a^	Y	No difference in the amount of improvement from pre- to post-test was found between the lighter ball group and the regulation ball group following 16 × 35-min practice sessions^g^. However, the lighter ball group performed better during match play, with more correct sets and a higher average daily success rate for the set and underarm serve

*N* no, *Y* yes

^a^Skill level of participants not specified

^b^In the Wright [22] study, the light balls were either a plastic “fun ball” (used for bowling, throwing and baseball hitting) or a polyethylene ball (used for basketball shooting). Conversely, the heavy balls were either a softball (used for bowling, throwing and baseball hitting) or a heavier than normal basketball (538 g). The baseball bats used were a light plastic bat (156 g) and a heavier little league bat (907 g)

^c^Movement clusters refer to the grouping of movement patterns for each individual based on the kinematic variables of interest. Lee et al. [27] adopted this analysis method to infer the number of movement solutions that children used to perform the task

^d^Larson and Guggenheimer [28] provided details regarding the coefficient of restitution for the two types of balls used in their study (i.e., the ratio of relative velocity of each ball after impact with the ground to the relative velocity of each ball before impact). The coefficient of restitution for the low compression balls ranged between 0.41 and 0.46, and for the standard balls it was between 0.53 and 0.58. Thus, we calculated that the low compression balls used in this study were likely to be similar to the balls used in other studies, which were described as being “75 % of the standard ball”

^e^Farrow and Reid [29] describe a “red” low compression ball as <50 % compression of the standard ball, while Buszard et al. [24] described a “red” low compression ball as 25 % compression of the standard ball. The balls used in these two studies were seemingly the same

^f^Hammond and Smith [7] do not describe the compression of the balls used in the study. However, the mass of the ball (46.08 g) indicates that it was similar to the “red” ball that was used in the Buszard et al. [24] study

^g^The final 6 days of practice in the Pellet et al. [10] study involved a match-play tournament, and all participants played with the regulation volleyball. Thus, the practice intervention, whereby the two groups practiced with different sized volleyballs, was only in fact 10 days in duration

### Psychological Factors

Five articles reported psychological benefits for children when using scaled equipment that simplified the task. For instance, 8-year-old children playing tennis with low compression balls on smaller courts reported more engagement during practice sessions compared with children playing with standard tennis balls on a full-size court [29]. The scaled condition created an environment that increased the number of viable opportunities to hit the ball, which consequently heightened engagement in the task. Children in the unscaled or full-size condition had fewer opportunities, which probably caused them to feel that the task was too difficult and to be less engaged. Children of a similar age have elsewhere reported preference for, and presumably greater engagement when, using scaled tennis equipment, including smaller racquets and lower compression balls [24] and lower nets [23]. In a basketball study involving 77 10-year-old children [9], 48 (62 %) preferred using a junior ball (as opposed to a women’s or men’s ball) and only seven (9 %) preferred using an adult men’s ball. Whilst the junior ball did improve shooting performance for all children, it was observed that shooting performance was significantly better when children used the ball of their preference, which was typically a ball smaller than the adult men’s ball.

Greater ‘shot-efficacy’, or the belief of a child that they have “the capacity to achieve the desired or expected effect from shooting” (p. 54) [12], has also been found in children playing basketball with a lighter ball [12] and a lower basket [17]. This was reported to be a consequence of the increased shooting success that children experienced when shooting in the modified conditions. Importantly, a heightened sense of skill mastery is considered to be an indicator of motivation for the task [32, 33]. The relationship is cyclical, as greater motivation tends to lead to greater physical activity levels, which in turn provides children with the opportunity to attain actual motor competence (or skill mastery). Significantly, actual motor competence is thought to be a strong predictor of physical activity in adolescent and adult years [34–37]. As such, it is possible that scaling the equipment and play area for children also contributes to future or ongoing participation in physical activity, which is inextricably linked to a number of health benefits, such as greater physical fitness and a reduced risk of obesity [38, 39].

### Skill Performance (and Acquisition) Factors

It has been well established that scaling equipment generates greater task success and better performance in a range of skills compared with unscaled or ‘adult’ equipment. For instance, in tennis, children playing with lower compression balls are able to strike the ball with greater ease [24, 25, 28]. Low compression balls bounce lower than standard tennis balls,^2^ allowing children to strike the ball in an optimal location relative to their height (i.e., waist height) [26]. Furthermore, children generate greater ball velocity whilst maintaining (or improving) hitting accuracy when using low compression balls,^3^ which indicates that children strike the softer ball with greater power and without the fear of the ball travelling too far [28]. In addition to these findings, it appears that performance is further enhanced when low compression balls are combined with scaled racquets [24]. However, results indicate that ball compression has a greater impact on hitting performance than racquet size, with the lowest compression balls generally producing the best performances.

Scaling the task for children also enhances skill learning opportunities during practice. Farrow and Reid [29] found that a combination of low compression balls and smaller court size increased the volume of practice in 8-year-old beginners, whereas practice with standard balls on a full-size court led to concomitant impairments in learning. The ‘adult’ practice conditions reduced the number of hitting opportunities, which effectively diminished chances for practice repetition and consequently learning. Furthermore, the combination of decreased hitting opportunities and a more difficult practice environment resulted in the children in the adult practice condition executing fewer successful forehands and backhands relative to the scaled conditions.

In a similar vein, other research has demonstrated that children (beginners to tennis) displayed the greatest improvements in a range of skills tests when using scaled racquets (17- and 24-in. racquets) compared with larger racquets (26-in. length racquets) following 16 sessions of practice [31]. Interestingly, the only skill in which performance with a larger racquet was commensurate with a smaller racquet was volleying, which may not be as influenced by the greater moment of inertia of a larger racquet. It is apparent that lighter racquets allow children to wield the racquet with greater ease, thereby facilitating the development of stroke-making ability.^4^

In addition to optimizing the practice environment, scaling equipment also leads to better performance during match-play conditions. For skilled children in tennis, low compression balls (compared with a standard ball) result in faster rallies, more shots played at a comfortable height (between hip and shoulder, as opposed to above the shoulder with the standard ball), and more shots played at the net [26]. In essence, playing with a low compression ball resulted in tennis match play that more closely resembled a professional adult match. Logically, if similar characteristics were observed in practice, it could be reasoned that this would lead to improved long-term outcomes for players learning the sport. A similar study with skilled children showed that lowering the net also had a positive influence on tennis match-play performance [23]. When the net was lowered from 0.91 to 0.67 m, children hit more shots at a comfortable height and in front of the baseline (which typically represents more aggressive play in tennis), and more volleys and winners.

Research in basketball also demonstrates the advantages of scaling equipment for children during match-play conditions. Arias and colleagues examined the effect of ball weight on children’s basketball match-play performance. Five of Arias’ studies [11–13, 15, 16] examined the same cohort of children,^5^ but the results suggested that children exhibited more dribbling and passing [14], increased shot frequency and greater shot success [12, 15], and a higher percentage of attempted lay-ups [11] when playing with a lighter ball (440 g) as opposed to a regulation ball (485 g) or a heavier ball (540 g). Additionally, the lighter ball resulted in more one-on-one situations, presumably because the lighter ball provided children with the opportunity to dribble and take on their opponents [16]. Similar results have also been reported in volleyball, with seventh grade girls displaying a higher percentage of successful sets and serves during match play when using a lighter ball (25 % lighter than a standard volleyball) [10]. In essence, these results are symptomatic of environments that have been constrained, via a lighter ball, to allow children to perform skills with greater success.

To summarise the skill performance (and acquisition) literature, it is apparent that children (a) perform skills better when the equipment and play area are scaled, (b) are presented with increased opportunities to practice skills, and (c) are able to play matches in a style that more closely resembles an adult match. Consequently, skill acquisition should be enhanced when children play sport in a scaled environment. However, no study has examined the influence of scaled equipment over a practice period longer than 8 weeks [31], so we cannot be certain that scaling equipment leads to greater learning in the long-term compared with the use of adult equipment. Future research programs need to place a major emphasis on longitudinal studies to provide a comprehensive analysis of the learning process.

### Biomechanical Factors

The primary argument of the constraints-led approach is that the body is biologically designed to discover and self-organize optimal movement patterns in response to the constraints imposed on the neuro-musculoskeletal system [42]. Thus, if a child plays tennis with a scaled racquet, their body will self-organize its movements in accordance with the constraints imposed by use of that particular racquet (whilst also within the boundaries of other task, environmental and organismic constraints). Indeed, it is evident across a number of studies that scaling equipment leads to the production of more functional movement patterns. For instance, when Buszard et al. [24, 25] asked children to perform a tennis forehand with low compression balls, two technical benefits were identified: the racquet was swung in a desirable low-to-high swing path and the ball was struck in front and to the side of the body.^6^ The benefits were most evident when children used the lowest compression ball of the three types tested, suggesting that a ball that bounced lower and travelled slower through the air provided children with an opportunity to adopt a more desirable technique. Likewise, in basketball, when the basket height was reduced, children adapted their movement patterns and shot with a slightly flatter trajectory [18]. Unfortunately, however, the results reported in this particular study provided no indication as to whether this adaptation was advantageous to shooting performance.

Significantly, a study involving 20 participants in four age groups—(a) 5–6 years, (b) 7–8 years, (c) 9–10 years and (d) 18–33 years—observed that throwing technique regressed when balls were used that were too large in relation to hand size [20]. Specifically, throwing technique showed most regression in the backswing and forearm components^7^ when the diameter of the ball exceeded the size of the participant’s hand width. Typically, participants adapted to the larger ball size by shortening their backswing, therein removing the ‘forearm lag’ by adopting a shot-put style of throw, and using two hands to control the ball. In comparison, participants displayed a more desirable throwing technique according to the fundamentals of overarm throwing when they were able to grasp the ball easily.

Similar results were also found when observing children’s catching performance. Seven-year-old children displayed a more mature catching style when attempting to catch a small ball compared with a large ball [21]. Indeed, children were more likely to catch the small ball cleanly in their hands without using their body for assistance. These findings have obvious ramifications for practitioners teaching throwing and catching, as children will require a smaller ball in order to perform these skills in a manner that is desirable for most sport and physical education settings.

There is also evidence that scaling equipment will reduce the risk of injury by constraining children’s technique to more efficient movement patterns. For example, shortening pitch length in cricket not only simplifies the skill for junior fast bowlers, but it also generates more efficient movement kinematics, particularly for younger bowlers [19]. Lower back stress fractures are very common in junior fast bowlers [43], and Elliott et al. [19] concluded that the shortened pitch length would decrease the likelihood of lower back injuries by reducing shoulder counter-rotation. Thus, constraining the task to optimize movement patterns ultimately has potential to reduce the risk of injury.

An interesting question is whether it is possible to quantify the amount of scaling required for each child, to allow desirable movement patterns to emerge. Gagen et al. [30] examined 4- to 10-year-old children who were required to perform a forehand hitting task in which they were instructed to “swing as hard as possible and hit the ball as closely to the centre of the racquet” as they could. Children performed this task using four different racquets that varied in length and mass. Gagen et al. [30] anticipated that the unique physical characteristics of each child (hand size, arm length, height, weight, functional leg length, grip strength, shoulder strength) would predict which racquet produced the most desirable performance, as measured by racquet-head speed and accuracy of contact on the racquet. The results showed that for each child one specific racquet produced better speed and accuracy than the other racquets; however, physical characteristics did not predict this ‘optimal’ racquet statistically. Thus, further research is required to understand the mechanisms underpinning the production of optimal movement patterns when using various equipment sizes.^8^

Finally, a novel approach to understanding the effect of equipment and play area modifications, among other constraints, on the performance of the tennis forehand was offered by Lee et al. [27]. In a point of difference from the other studies critiqued in this review, their approach focused on creating a variable practice environment by constantly manipulating key task constraints, including net height and court size. Children exposed to these practice conditions, in what was termed the non-linear pedagogy group, achieved similar skill improvements but with greater degeneracy in their movement patterns than the linear pedagogy group (in which children used the one size of equipment in an environment that emphasized repetition). The authors interpreted this disparity in degeneracy to mean that the children in the non-linear group discover more movement strategies to achieve the task goal. However, the children who used the one size of equipment and participated in more traditional practice settings (the linear group) rated better than their counterparts on an assessment of forehand technique fundamentals, which in turn, might cause practitioners to contemplate the importance of form versus function. Significantly, in the context of this review, this study chose not to detail the timing or type of scaled equipment that was used, therein clouding direct comment on the efficacy of specifically scaled constraints. Nevertheless, the findings do provide a novel method of modifying the equipment and play area to facilitate the self-organization of movement patterns, which might prove a fertile area of future scaling research.

### Cognitive Processing Factors

A well-established phenomenon within the motor learning literature is that cognitive processes influence skill acquisition and performance. Acquiring skills with heightened conscious involvement, characterized by the attempt to consciously discover verbal rules about the skill, is referred to as explicit motor learning [46]. Comparatively, acquiring skills via sub-conscious processes, whereby the learner has difficulty verbalizing the step-by-step processes of the skill’s performance, is referred to as implicit motor learning [47, 48]. Research over the past 2 decades has consistently shown that implicit acquisition of motor skills is more advantageous than explicit learning when performance is subsequently required in environments that induce psychological stress [47, 49] or physiological fatigue [50, 51]. Furthermore, dual-task transfer tests have shown that individuals who have acquired a skill implicitly are able to simultaneously perform a cognitively demanding secondary task whilst performing the motor skill [52–54]. In contrast, individuals who acquire a skill explicitly typically have difficulty multi-tasking in these transfer tests.

Several practice methods have been identified that encourage implicit motor learning. Most relevant to this review is the concept of ‘errorless’ or ‘error-reduced’ practice. Research across a range of skills demonstrates that when errors are infrequent during practice, skills are acquired with minimal reliance on cognitive resources (i.e., working memory); thus, implicit learning benefits are evident [53–58]. Given that scaling equipment simplifies skills for children, thereby increasing success experienced, it can be reasoned that scaling will place fewer demands on working memory and, therefore, encourage implicit motor learning.

This hypothesis was recently examined using a dual-task methodology to measure children’s skill performance when attention resources were occupied by a secondary task [25]. Children performed a basic tennis-hitting task in two attention conditions (single-task and dual-task) using two types of equipment (scaled and full size). The scaled equipment included a lower compression ball and a smaller racquet (23-in. length), whereas the full-size equipment included a standard tennis ball and an adult-sized racquet (27-in. length). Results showed that hitting performance and hitting technique were better when scaled equipment was used, demonstrating that scaled equipment did indeed simplify the skill for children. For the less skilled children in the study, hitting performance was not disrupted by a cognitively demanding secondary task when using scaled equipment. However, performance deteriorated significantly when full-size equipment was used, suggesting that equipment that increases skill difficulty places larger demands on working memory resources than equipment that does not (i.e., scaled equipment). While this study only assessed conscious processes during performance on a small number of trials (as opposed to a learning design), the results corroborate the prediction that modification of equipment to simplify a skill reduces conscious processing.

The influence of equipment modification on conscious processing can also be inferred from studies with adults. A golf putter designed to increase skill difficulty resulted in greater preparation time prior to skill execution, which the authors interpreted to represent greater conscious processing [59]. Similarly, equipment that increased skill difficulty demanded greater attention resources during movement preparation and movement execution [60]. Thus, consistent with the findings of Buszard et al. [25], equipment that increases skill difficulty (e.g., full-size equipment for children) places heavy demands on attention resources, thereby leading to a more explicit control of motor performance.

Interestingly, a similar hypothesis regarding equipment modification was expressed over 40 years ago. In a study that examined the acquisition of throwing skill, Egstrom et al. [61] explained, “the adjustments made during the practice periods while learning to throw the light ball accurately resulted in automatic adaptations at a subconscious level. When the subjects then transferred to the heavy ball after a period of practice, the increased weight could have elicited a response … which in turn brought the impulse to consciousness…” (p. 424). Hence, throwing with a lighter ball seemingly encouraged implicit motor learning, whereas the heavier ball more likely activated explicit processes.

## Limitations and Future Directions

We have outlined six major limitations of the literature reviewed. These limitations should guide directions for future research.

### Only Five Studies Have Assessed ‘Learning’

**Fig. 3:**
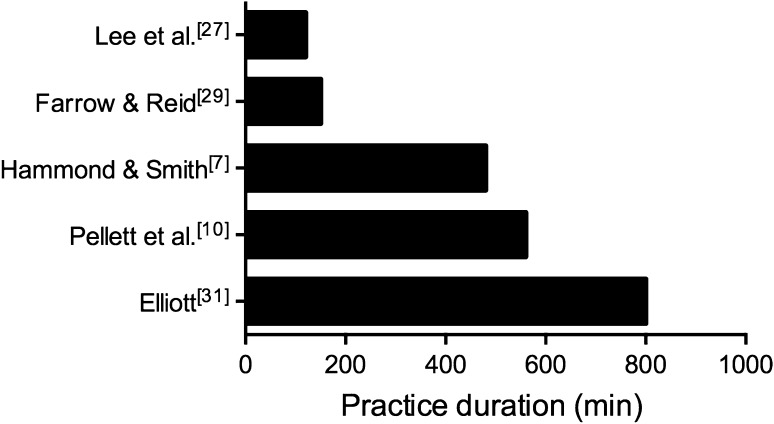
The total practice duration (min) for the five studies that examined the influence of equipment scaling on skill acquisition over a period of practice. There has been a trend for shorter studies over the past 25 years

Of the 25 studies examined, only five assessed the influence of equipment modification on learning over a period of time, with interventions ranging from 5 to 16 sessions of practice [7, 10, 27, 29, 31] (see Fig. 3). Two of these studies reported learning advantages when children were exposed to a scaled environment [29, 31]. Whilst this highlights the positive impact that scaling can have in such a short period of time, two other studies found no differences in the amount of skill improvement when scaled or adult equipment were used [7, 10]. However, these latter studies failed to control for age or skill level [7] or biased ‘adult’ equipment in the skill testing protocol [10]. The fifth study did not actually examine the impact of equipment scaling, but rather the effect of constantly manipulating the equipment and play area throughout practice [27]. It is therefore clear that longitudinal studies are needed to provide a comprehensive analysis of skill learning associated with equipment scaling. Currently, the lengthiest intervention is 8 weeks (16 sessions) and this study was conducted 3 decades ago [31]. In addition to short practice interventions, no study has assessed whether equipment and/or play area scaling leads to the development of motor skills that can be adapted to situations that differ from the practice. Similarly, only one study assessed skill retention following a period of practice, which was measured 1 week after the post-test [27]. Given that measures of skill transfer and skill retention are often considered more insightful assessments of motor learning than accelerated performance gains [62], we are limited in our conclusions regarding the influence of scaling on children’s motor skill acquisition.

### Skills Tests Have Often Involved Few Trials and Lacked Ecological Validity

The tests used to measure skill performance also need to be scrutinized. Many studies have assessed skill on the basis of a small number of trials, presumably for logistical reasons. For example, studies assessing equipment scaling in tennis have made inferences about skill performance based on 6–15 strokes [7, 24, 25, 27, 31]. Likewise, basketball studies have assessed shooting performance based on five shots [8, 9]. Although statistical differences between scaling conditions have typically been found when using such a small number of trials, future research should seek to include more test trials to allow analysis of learning effects and examination of movement variability.

Additionally, a challenge for researchers measuring skill is to design tests that are well controlled and ecologically valid; however, these two factors are often difficult to reconcile. For example, tennis studies have typically required children to strike an incoming ball of controlled velocity and trajectory (e.g., via an underarm throw or a ball machine) as a measure of their competence [24, 25, 30, 31]. The assumption is that children who display better performance at this task will also perform better during match conditions. However, given that a ball being projected from an underarm throw or a ball machine is not representative of match conditions, we cannot be certain of this assumption. In contrast, skills tests adopting methods such as ‘rallying’ in tennis provide an ecologically sound alternative. Such measures, however, appear to be difficult to employ with young novice children, because of the increased difficulty of performing skills within a match context [29]. As such, designing tests that are well controlled and ecologically valid will continue to pose a challenge for researchers. Some studies have assessed skill by examining performance during match conditions [11–16, 23, 26]. These assessments are ecologically valid, but they provide a difficult assessment of ‘learning’ because of the number of factors that can influence match-play performance. Future research should therefore look to incorporate multiple measures of skill performance that range from ecologically sound assessments to laboratory style tests with a large number of test trials.

### Greater Exploration of Equipment Constraints is Required

For logistical reasons, most studies have examined the influence of scaling equipment on performance by manipulating one specific variable (e.g., basketball mass or racquet length) and comparing this against its full-size counterpart. Although this experimental approach has provided the basis for understanding equipment scaling in children’s sport, there are many variables that influence equipment’s haptic properties. Additionally, equipment can influence a child in different ways, depending on their intrinsic dynamics (e.g., age, skill level, body composition). For instance, the influence that a basketball has on a child’s performance will be largely dependent upon the mass and diameter of the ball and the child’s maturational, physical and skill development. We acknowledge that for logistical reasons it may be impractical to examine every variable related to both equipment and the performer; however, studies should seek to offer children more equipment alternatives in an attempt to discover which is most appropriate for each child. Similarly, scaling should not necessarily be limited to one variable, as it is likely that the combination of scaled constraints will produce the most desirable results [17, 23, 24, 27, 29]. Indeed, in practice, this is invariably what materializes, with sport federations like the International Tennis Federation and the International Basketball Federation employing combinations of scaling to expedite learning.

Additionally, in an attempt to discover the most optimal scaling ratio, researchers should consider applying concepts from the body-scaling literature, namely pi ratios. This refers to the ratio between a metric of an actor and a metric of an action space [63]. For example, when required to pick up cardboard cubes, children change from one-hand grasping to two-hand grasping when the size of the cube (metric of action space) exceeds a certain threshold relative to children’s finger span (metric of the actor) [64]. In a similar vein, the movement patterns that children produce when playing sport may be dependent upon the pi ratio for the given task. The throwing study by Burton et al. [20] speaks to this, whereby throwing technique regressed when the diameter of the ball (metric of action space) increased to a size that was larger than the participant’s hand (metric of actor). Accordingly, the pi ratio offers a practical and seemingly under-utilized means to quantify the most beneficial scaling ratio on the basis of individual characteristics.

### Studies Examining Skilled Children are Scarce

The majority of studies have assessed children with limited skill. From an experimental perspective, this provides researchers with an opportunity to clearly identify the effect that equipment scaling has on children’s performance and learning without the influence of prior experience confounding the results. Whilst these studies have provided the framework for future research, there is a clear need to examine children who possess a degree of skill within the task.^9^ Therefore, future research should investigate the interaction between equipment scaling, learning and the skill level of children, as this will assist practitioners in deciding when children should progress from scaled equipment to adult equipment.

Indeed, determining when children should progress from scaled to adult equipment is a challenge as coaches and teachers often work with groups of children, which make it difficult to progress children on an individual basis based on skill development. Additionally, changing from scaled to adult equipment is likely to demand a recalibration of coordinative movement patterns and the associated perceptual processes [65]. It has been reported that children regress from a mature movement pattern to a less mature movement pattern when switching from a light to a heavy implement when performing a striking task [66]. As such, it is important that practitioners consider carefully the progression from scaled to adult equipment.

As an example of how sports organizations can address the progression dilemma, the International Tennis Federation developed a three-stage system, which children are encouraged to progress through before using adult equipment. There is a “red” stage (5–8 years), an “orange” stage (7–9 years) and a “green” stage (8–10 years). For each stage, there are recommended guidelines for the required tennis skill competence that children should display. Whilst this system still requires empirical evidence to support each of its recommendations, it does provide reasonable guidance for practitioners in the interim.

### Only One Study Has Assessed the Theoretical Underpinnings of Equipment Scaling

Two theoretical frameworks for assessing equipment scaling have been discussed in this review. Proponents of the constraints-led framework argue that scaling task constraints to simplify a skill will encourage a sub-conscious mode of learning by allowing children to search for the most optimal solution [3]. Likewise, proponents of implicit motor learning theory argue that scaling equipment to simplify a skill is likely to encourage learning via sub-conscious processes; however, implicit motor learning theorists argue this for different reasons than the constraints-led theorists (see Sect. 3.6) [25]. It is apparent that researchers need to clarify the nuances between the *sub*-*conscious* exploration of movements that result in greater functional movement variability (as outlined by constraint-led theorists), and the *conscious* search for new solutions to the movement pattern, resulting in accumulation of task-specific declarative knowledge about the skill (as outlined by implicit motor learning theorists). We argue that scaling equipment is more likely to discourage conscious exploration of a motor solution because of the accumulation of fewer errors, thereby diminishing engagement with working memory [25]. Our argument is best summarized by Lam et al. [60] when describing ‘self-organization’ during errorless practice; however, replace the term *errorless learning* with *scaling equipment*: “Ironically, errorless learning [*scaling equipment*] appears to result in motor performance that makes only limited demands on attention, implying that solving the motor problem, as Bernstein [67] described it, is more an implicit process than an explicit process” (p. 1553). In laypersons’ terms, it is more likely that children will solve the motor solution and develop coordinative structures via implicit processes when using equipment that simplifies the skill. Conversely, it is more probable that explicit processes will intervene when using equipment that increases skill difficulty and consequently circumscribes movements.

### More Multidisciplinary Research is Needed

It is apparent from this review that scaling in children’s sport influences multiple factors that have a role in motor skill acquisition. Thus, in order to further our understanding of this issue, multidisciplinary research is required in which experts from a variety of disciplines provide a unique perspective on the findings that will ultimately offer a holistic approach to skill acquisition. A few of the studies in the review incorporated findings from multiple disciplines; however, more is required if we wish to elucidate the underlying mechanisms influencing the acquisition of motor skills.

## Conclusion

Despite the aforementioned limitations in the literature reviewed, most of the evidence suggests that the scaling of equipment and play area in children’s sport is beneficial to motor skill acquisition. These benefits include greater engagement with and enjoyment of the task, enhanced performance of skills, expedited skill improvements (although the need for a longitudinal study of greater than 8 weeks is paramount), improved match performance (closer resemblance to an adult match), the development of more desirable movement patterns and increased likelihood of learning and performing implicitly. The next step in this body of research is to substantiate the progression of scaling as age increases and skill level develops. Indeed, the critical skill for a practitioner is to know how and when to progress a child from scaled equipment to adult equipment. Currently, as illustrated in Fig. 1, many sports authorities recommend children (i.e., by the age of 10) play on adult-sized fields well before reaching their adult height. Whilst we acknowledge that children’s height is not the only variable that should be considered when determining the optimal scaling ratio, the figure does highlight the need for more appropriate guidelines. Furthermore, it is important to know which constraints of the task should be scaled in order to maximize the benefits. For instance, in tennis, scaling the court appears more advantageous than modifying the ball, but using low compression balls seems better for performance than scaling the racquet.

Most of the studies in this review were focused on tennis and basketball and, although these findings can be generalized to other settings, there is a clear need for researchers to explore scaling in other sports. By way of example, most sports now endorse modified junior programs; yet, given that research is scarce in most sports, these programs are seemingly based on limited empirical evidence. Nonetheless, given the findings of this review paper, sports organizations and physical educators should continue to encourage and develop junior modified sport programs. Whilst scaling equipment for children will continue to be challenged by practitioners who want children to begin playing the adult game from a young age, the literature clearly shows that children will actually play sport in a manner that more closely resembles adult performance when using scaled equipment. It is evident that all children should have access to appropriately scaled equipment when playing sport, which should sustain participation and enhance skill acquisition.

## Footnotes

1 The Cochrane Collaboration tool for assessing risk of bias also includes *performance bias*. This refers to the blinding of participants to the allocated intervention. Whilst this is important for healthcare research, this is irrelevant to research examining scaling in children’s sport, as children will always be aware of the experimental group/condition that they are exposed to by virtue of participating in the study (e.g., children will know that they are in the scaled equipment group when they are provided with the scaled equipment). As such, we have not included assessment of *performance bias* in this review.

2 Tennis ball rebound heights are examined by dropping a ball from 254 cm and measuring the subsequent height of the first bounce. According to the International Tennis Federation’s recommendations for ball specifications [40], a ball that is 25 % compression of a standard tennis ball bounces 90–105 cm, a ball that is 50 % compression bounces 105–120 cm, a ball that is 75 % compression bounces 120–135 cm, and a standard tennis ball bounces 135–147 cm.

3 Larson and Guggenheimer [28] measured ball velocity for 7- to 9-year-old children when rallying with a professional coach. Results revealed that ball velocity was on average 6.5 km/h faster when using the low compression balls (50 % compression) than the standard balls.

4 Beak et al. [41] showed that children are sensitive to changes in moment of inertia when they swing tennis racquets with their vision occluded (i.e., when the superficial information about the racquet is not available). This is in contrast to adults who demonstrated an ability to detect small changes in moment of inertia whether vision was occluded or not. The authors concluded that this highlights the need for children to be exposed to a broad range of racquets that vary in moment of inertia. This study was not included in the review as it was published in a book chapter (i.e., not in a peer-reviewed scientific journal).

5 It was apparent that five of the six studies published by Arias and colleagues in this review were based on data from one cohort of participants, as evidenced by the same participant details and almost identical methodology sections across the studies.

6 It is acknowledged that these measures were derived from a two-dimensional biomechanical measurement.

7 Burton et al [20] analyzed throwing technique based on Robertson’s [44] recommendations, which included five components of the overarm throw: (1) backswing, (2) humerus, (3) forearm, (4) trunk and (5) feet.

8 Similar results to Gagen et al. [30] have been reported elsewhere. Buszard et al. [24] included children’s height as a covariate when analyzing the influence of equipment scaling on tennis performance; however, height did not have a significant influence on the results. Likewise, Chase et al. [17] reported low correlation values between basketball shooting performance and anatomical measures (height, hand width and hand length) for both girls and boys (*r* ranged from 0.12 to 0.29). Conversely, in a study that examined shaft flexibility in golf clubs for junior golfers, it was observed that 21 of 30 participants displayed best performance with one particular shaft, and this “best” shaft was most influenced by the child’s strength, arm span and golf experience [45].

9 Nine of the studies reviewed did investigate skilled (or relatively skilled) children [11–16, 23, 26, 28]; however, five of Arias’ studies involved the same cohort of participants. Also, despite the apparent advantages for skilled children playing sport with scaled equipment, there was no measure of learning in these studies.
